# Huaier Polysaccharide Alleviates Dextran Sulphate Sodium Salt-Induced Colitis by Inhibiting Inflammation and Oxidative Stress, Maintaining the Intestinal Barrier, and Modulating Gut Microbiota

**DOI:** 10.3390/nu16091368

**Published:** 2024-04-30

**Authors:** Yi-Fei Tang, Wen-Yin Xie, Hong-Yu Wu, Hai-Xiang Guo, Fan-Hao Wei, Wen-Zhi Ren, Wei Gao, Bao Yuan

**Affiliations:** 1 Department of Laboratory Animals, College of Animal Sciences, Jilin University, Changchun 130062, China; tangyf9921@mails.jlu.edu.cn (Y.-F.T.); wyxie22@mails.jlu.edu.cn (W.-Y.X.); wuhy21@mails.jlu.edu.cn (H.-Y.W.); hxguo23@mails.jlu.edu.cn (H.-X.G.); weifh22@mails.jlu.edu.cn (F.-H.W.); renwz@jlu.edu.cn (W.-Z.R.); 2 Changchun National Experimental Animal Center, Jilin University, Changchun 130062, China

**Keywords:** ulcerative colitis, Huaier polysaccharide, oxidative stress, gut microbiota, gut barrier

## Abstract

The incidence of ulcerative colitis (UC) is increasing annually, and UC has a serious impact on patients’ lives. Polysaccharides have gained attention as potential drug candidates for treating ulcerative colitis (UC) in recent years. Huaier (*Trametes robiniophila* Murr) is a fungus that has been used clinically for more than 1000 years, and its bioactive polysaccharide components have been reported to possess immunomodulatory effects, antitumour potential, and renoprotective effects. In this study, we aimed to examine the protective effects and mechanisms of Huaier polysaccharide (HP) against UC. Based on the H_2_O_2_-induced oxidative stress model in HT-29 cells and the dextran sulphate sodium salt (DSS)-induced UC model, we demonstrated that Huaier polysaccharides significantly alleviated DSS-induced colitis (weight loss, elevated disease activity index (DAI) scores, and colonic shortening). In addition, HP inhibited oxidative stress and inflammation and alleviated DSS-induced intestinal barrier damage. It also significantly promoted the expression of the mucin Muc2. Furthermore, HP reduced the abundance of harmful bacteria *Escherichia-Shigella* and promoted the abundance of beneficial bacteria *Muribaculaceae_unclassified*, *Anaerotruncus*, and *Ruminococcaceae_unclassified* to regulate the intestinal flora disturbance caused by DSS. Nontargeted metabolomics revealed that HP intervention would modulate metabolism by promoting levels of 3-hydroxybutyric acid, phosphatidylcholine (PC), and phosphatidylethanolamine (PE). These results demonstrated that HP had the ability to mitigate DSS-induced UC by suppressing oxidative stress and inflammation, maintaining the intestinal barrier, and modulating the intestinal flora. These findings will expand our knowledge of how HP functions and offer a theoretical foundation for using HP as a potential prebiotic to prevent UC.

## 1. Introduction

Ulcerative colitis (UC) is an inflammatory bowel disease that affects the sigmoid colon and rectum, and its incidence is increasing annually; UC seriously impacts patients’ lives [1]. The development of UC is linked to four factors: immunology, the intestinal barrier, genetics, and the environment [2]. The course of UC is characterised by a “relapse-remission” pattern, which requires different therapeutic approaches to induce and maintain remission. There is no cure for UC, so the goals of current treatment are to alleviate symptoms, improve quality of life, and prevent and treat complications. Pentaaminosalicylic acid [3] and immunosuppressants [4] are commonly used to treat UC, but long-term treatment can cause severe side effects. Thus, the development of a reliable and secure dietary supplement is crucial for the mitigation and prevention of UC.

Natural polysaccharides are abundant in plants, animals, and microbes and originate from a variety of sources. Several studies have shown that dietary supplementation with polysaccharides has multiple benefits for the body. Fuzhuanbrick tea is a post-fermented tea (black tea) fermented by complex microorganisms [5]. Previous research has reported that there are many chemical components in Fuzhuanbrick tea, including flavan-3-ols and their derivatives, phenolic acids and their derivatives, flavones and their glycosides, terpenoids, alkaloids, and tea polysaccharides [6]. Fuzhuanbrick tea polysaccharide may enhance DSS-induced ulcerative colitis by modulating intestinal tryptophan metabolism [7]. By modifying intestinal contraction, *Lycium barbarum* polysaccharide enhances glucose metabolism in diabetic mice [8]. *Scutellaria baicalensis* polysaccharide treatment promoted the relative abundance of Bifidobacterium and Lactobacillus in the gut microbes of mice and increased the expression of intestinal TJ proteins, thereby alleviating DSS-induced colitis [9]. Huaier polysaccharides have drawn a lot of attention lately due to their potential to treat cancer [10]. Many investigations have demonstrated that Huaier polysaccharide can inhibit the growth of many cancers, such as liver cancers [11], breast cancers [12], and gastrointestinal cancers [13]. However, there are fewer studies on the mitigating effect of Huaier polysaccharide on colitis.

For over a millennium, Huaier (*Trametes robiniophila* Murr) has been utilised in medicinal settings [14]. Huaier contains a variety of components, including polysaccharides, proteins, ketones, and alkaloids, whereby proteoglycans and polysaccharides are thought to be the primary bioactive components [15]. Previous studies have shown that Huaier extract has immunomodulatory activity [16,17,18] and can exert a protective effect on cells through the activation of autophagy [19], the inhibition of ferroptosis [20], the inhibition of endoplasmic reticulum stress [21], and other processes. Huaier aqueous extract has been demonstrated to alleviate dextran sulphate sodium salt (DSS)-induced intestinal injury and inflammatory response by inhibiting NLRP3 inflammatory vesicle activation [22] and inhibiting colon tumorigenesis induced by the combination of DSS and azoxymethane (AOM) [23]. Natural polysaccharides may be potential prebiotics, but whether HP can alleviate gut barrier damage induced by DSS and the effects of HP on gut microbiota and gut metabolism are unknown.

DSS-induced colitis was commonly used as a model to simulate ulcerative colitis with some symptoms similar to human UC [24]. Meanwhile, the H_2_O_2_-induced HT-29 oxidative stress model was often used as an in vitro model of human intestinal function to study cellular oxidative stress [25,26]. Therefore, in this study, we evaluated the protective effect of HP in vitro using an H_2_O_2_-induced HT-29 cell injury model and assessed the protective influence of dietary HP supplementation against colitis utilizing a DSS-induced ulcerative colitis mouse model. We explored the impact of HP on the mechanical barrier of the intestines, oxidative stress, and the inflammatory response. Additionally, we employed 16S rRNA sequencing and untargeted metabolomics to analyse the impact of HP on the gut microbiota and metabolism. These findings will expand our knowledge of how HP functions and offer a theoretical foundation for using HP as a dietary supplement to prevent UC.

## 2. Materials and Methods

### 2.1. Antibodies

Affinity Biosciences (Cincinnati, OH, USA) provided the anti-Claudin 1 antibody, the anti-Muc2 antibody, the anti-Occludin antibody, and the anti-ZO-1 antibody. Cell Signaling Technology (Danvers, MA, USA) provided the anti-GAPDH antibody and anti-rabbit IgG.

### 2.2. Cell Culture

HT-29 cells (CX0075, Boster, Wuhan, China) were cultured in DMEM supplemented with FBS (10%) and penicillin–streptomycin (1%). The culture conditions were 37 °C. The carbon dioxide content was 5%. Cells were passaged when the confluence reached 80–90%. The cells were free of mycoplasma or bacterial contamination.

### 2.3. CCK-8 Assay

Huaier polysaccharide (HP) was purchased from Yuanye Bio (S28138, Shanghai, China). Using the CCK-8 assay, the cytotoxicity of HP was examined with the concentration gradients of HP set at 0, 50, 100, 200, 400, 800, and 1600 μg/mL. Cells were obtained by trypsin digestion and centrifugation when the cell confluency reached 80–90%. Then, a 96-well plate was filled with 10,000 cells per well. The cells were fed with DMEM media containing 10% FBS at various doses of HP the next day, then after twenty-four hours, a serum-free medium containing ten percent CCK-8 detection reagent was added for two hours. With the help of an enzyme marker (TECAN, Männedorf, Switzerland), the absorbance at 450 nm was determined.

### 2.4. Apoptosis Assay

When the cell confluence of the six-well plate reached 60%, the HT-29 cells were pretreated with 400 μg/mL, 800 μg/mL, or 1600 μg/mL HP for twenty-four hours and then treated with 800 μM H_2_O_2_ for four hours. The cells were stained in accordance with the manufacturer’s instructions using the Annexin V-FITC/PI apoptosis kit (Liankebio, Hangzhou, China). Apoptosis was then evaluated using flow cytometry (Beckman, Miami, FL, USA). 

### 2.5. Reactive Oxygen Species (ROS) Assay

When the confluence of the six-well plate reached 60%, the HT-29 cells were pretreated with 400, 800, or 1600 μg/mL HP for 24 h. The probe was loaded using the ROS kit (MA0219, Meilunbio, Dalian, China) as instructed by the manufacturer, and the cells were subsequently treated with 800 μM H_2_O_2_ for 4 h. Trypsin digestion was subsequently performed to collect the cells for the flow cytometry assay.

### 2.6. Animal Experiments

Twenty-four SPF-grade male BALB/c mice were obtained from Liaoning Changsheng (Benxi, China). All the experimental animals were housed in the Specific Pathogen-Free Animal Barrier Facility of the Jilin University Laboratory Animal Centre with a 12 h light/12 h dark cycle and free access to distilled water and the same food. After a 7-day period of over-acclimatisation, 24 mice were allocated randomly to three groups: the control group (NC), the DSS group (DSS), and the HP group (DSS + HP) (*n* = 8). The NC and DSS groups were given saline by gavage every day for 0–14 days, while the HP intervention group was treated with 200 mg/kg HP by gavage daily (three concentrations of 50 mg/kg/d, 100 mg/kg/d, and 200 mg/kg/d were employed in the pretest, according to the literature) [27,28,29]. The 14–21-day-old NC group mice were provided normal sterile water, while mice in the DSS and HP intervention groups were provided sterile water supplemented with 3% DSS (MP Biomedicals, CA, USA); the mice in each group were scored daily during this period to determine the DAI score [30]. The mice were fasted overnight, and samples were collected on the 22nd day. The mice were anaesthetised using isoflurane and then euthanised using cervical dislocation. Blood samples were spun for ten minutes at 3000 rpm, and the supernatant was then removed and kept in a freezer set at −80 °C; part of the colon sample was placed in 4% paraformaldehyde for fixation, after which the remaining colon samples were homogenised into 3 portions and kept in a freezer at −80 °C. The contents of the cecum were flash-frozen in liquid nitrogen for 1 h and subsequently stored at −80 °C. The experimental protocol was reviewed and approved by the Laboratory Animal Welfare and Ethics Committee of Jilin University (SY202305009), and approval was granted on 3 August 2023. We made every effort to minimise animal suffering and the number of animals sacrificed.

### 2.7. Evaluation of Disease Activity Index

To evaluate the severity of colitis, the Disease Activity Index (DAI) score is frequently utilised [31]. The Disease Activity Index (DAI) in this study was calculated using the formula: DAI = Faecal Trait Score + Faecal Blood Score + Weight Loss Score. Based on previous studies [32], we used the following scores: weight loss: 0 (0%), 1 (1–5%), 2 (5–10%), 3 (10–20%), and 4 (>20%); faecal consistency: 0—normal, 2—loose and unformed faeces, and 4—liquid faeces; and rectal bleeding: 0—normal, 2—significant bleeding, and 4—major bleeding.

### 2.8. Enzyme-Linked Immunosorbent Assay (ELISA)

To homogenise the colon tissues, a Jingxin tissue grinder (Shanghai, China) was used, and the homogenisation medium contained PBS (9 mL of PBS per 1 g of tissue). The resulting homogenate was spun for ten minutes at 3000 rpm. An ELISA kit from SINOBESTBIO (Shanghai, China) was utilised to assess the levels of IL-6, IL-1β, TNF-α, SOD, MDA, and T-AOC in the serum. Additionally, the levels of IL-6, IL-1β, TNF-α, LPS, MPO, SOD, MDA, and T-AOC in the supernatant of the colon tissue homogenates were determined using an ELISA kit.

### 2.9. Histopathological Staining

The fixation of colon tissue using 4% paraformaldehyde and paraffin was embedded according to routine procedures. Paraffin sections 5 μm in thickness were subjected to haematoxylin–eosin (HE) staining, alcian blue (AB) staining, and periodic acid–Schiff (PAS) staining. Paraffin slices of 5 μm were utilised for pathological staining. Among them, haematoxylin–eosin (HE) staining was used to observe the morphology and pathological changes in the colon; the glycoproteins released by goblet cells were observed using alcian blue (AB) and periodic acid–Schiff (PAS) staining.

### 2.10. Western Blotting and Immunohistochemistry

To assess the expression of Occludin, Claudin 1, and ZO-1 in colon tissues, Western blot analysis was performed. Using a BCA kit, the protein concentration was ascertained after the colon tissue’s total protein was extracted using RIPA. Proteins were isolated using 10% SDS-PAGE and were then wet processed onto PVDF membranes. The membrane was blocked for 1 h at room temperature; the primary antibody was added, and the sample was incubated for 2 h at room temperature with a primary antibody dilution ratio of 1:1000. The membrane was washed three times for ten minutes each time. After adding the secondary antibody, the samples were incubated at room temperature for one hour at a dilution ratio of 1:5000; the membrane was then washed 5 times for 5 min each, and the ECL (SW133-01, Seven, Beijing, China) was used for imaging observation.

Immunohistochemistry was used to determine the Muc2 protein expression in colon tissue sections. The primary antibody was diluted at a ratio of 1:200 and analysed using an immunohistochemistry kit (Boster, Wuhan, China).

### 2.11. 16 S rRNA Sequencing

The CTAB method was used to extract the caecal content DNA. The PCR amplification of the V3 + V4 region (341F: 5′-CCTACGGGGNGGCWGCAG-3′, 805R: 5′-GACTACHVGGGGTATCTAATCC-3′) was carried out. Two percent agarose gel electrophoresis was used to recover and purify the PCR-amplified products, and an Agilent 2100 bioanalyzer was used to evaluate the library’s quality; the quality of the library was assessed using an Agilent 2100 bioanalyzer (Santa Clara, CA, USA). Bipartite sequencing of 2 × 250 bp was performed using a NovaSeq 6000 sequencer. To process the sequencing data, several tools, including Vsearch, fqtrim, FLASH, and cutadapt, were used. Subsequently, the LianChuan BioCloud platform (https://www.omicstudio.cn/ accessed on 22 September 2023) was utilised for bioinformatics analysis.

### 2.12. Nontargeted Metabolomics

In total, 100 mg of liquid nitrogen-milled cecum content was weighed out and put into an EP tube along with 500 μL of an 80% methanol aqueous solution. The supernatant was centrifuged at 15,000× *g* for 20 min at 4 °C for 5 min. The supernatant was placed in a new EP tube, and the final concentration of methanol was 35% with the addition of mass spectrometry-grade purified water. The Vanquish UHPLC system from Thermo Fisher (Waltham, MA, USA) in Germany was utilised to perform UHPLC–MS/MS analyses. The mobile phases were 0.1% formic acid and methanol in positive mode and 5 mM ammonium acetate and methanol in negative mode. The data were loaded into Compound Discoverer software (version 2.0, Thermo Scientific) for screening, molecular formula prediction using molecular ion peaks and fragment ions, and comparison with the mzCloud (https://www.mzcloud.org/ accessed on 22 September 2023), mzVault, and Masslist databases. The scanning range was set to *m*/*z* 100–1500. Using blank samples allowed for the removal of background ions. After normalizing the quantitative results, the data were examined through analysis.

### 2.13. Statistical Analysis

The mean ± standard deviation was utilised to present the experimental data. Statistical analysis and plotting were performed with GraphPad Prism 9.4 (La Jolla, CA, USA). Multiple groups were compared using a one-way ANOVA followed by Dunnett post hoc test. *p* < 0.05 was considered to indicate statistical significance.

## 3. Results

### 3.1. HP Alleviates H_2_O_2_-Induced Cellular Damage

**Figure 1:**
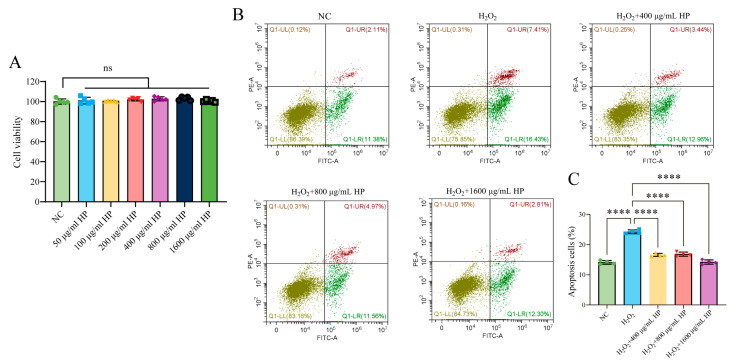
HP alleviates H_2_O_2_-induced apoptosis. (**A**) CCK-8 assay was performed to analyse the cytotoxicity of HP. (**B**) Flow cytometry was used to detect the inhibitory effect of HP on H_2_O_2_-induced apoptosis. (**C**) Quantification of apoptosis rate of cells in different treatment groups. (*n* = 3–5) **** *p* < 0.0001, ns *p* > 0.05.

**Figure 2:**
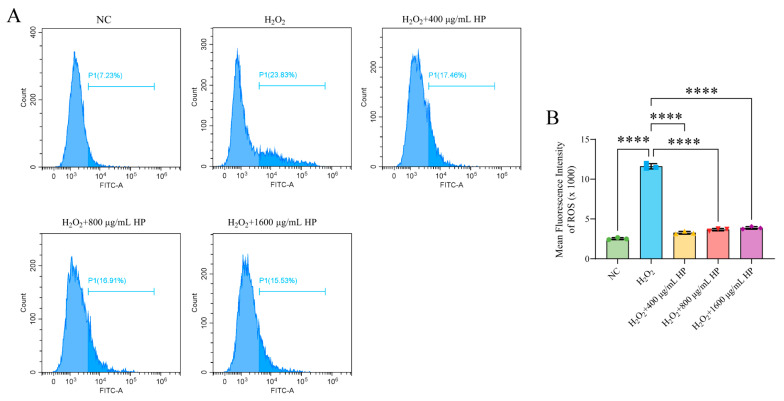
HP alleviates H_2_O_2_-induced ROS generation. (**A**) Flow cytometry was used to analyse the inhibitory effect of HP on H_2_O_2_-induced ROS generation. (**B**) Quantitative analysis of the mean fluorescence intensity was performed for each group. (*n* = 3) **** *p* < 0.0001.

To determine the optimal HP concentration for use in cellular experiments, HT-29 cells were exposed to a concentration gradient of 50–1600 μg/mL for 24 h. The results of the CCK-8 experiment showed that HP at 50–1600 μg/mL had no effect on cell proliferation (*p* > 0.05) (Figure 1A). The effects of HP pretreatment on H_2_O_2_-induced apoptosis and ROS generation were evaluated using an H_2_O_2_-induced oxidative stress model. The results showed that 400, 800, and 1600 μg/mL HP significantly inhibited H_2_O_2_-induced apoptosis (Figure 1B,C) and ROS generation (Figure 2A,B) (*p* < 0.001).

### 3.2. HP Relieves the Symptoms of DSS-Induced Colitis 

**Figure 3:**
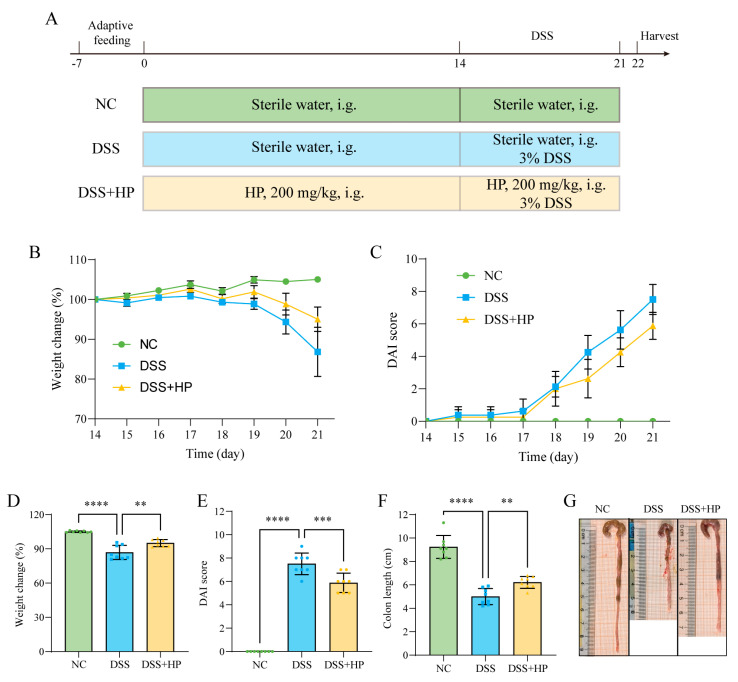
HP inhibited DSS-induced colitis-like symptoms. (**A**) A schematic diagram of the experimental design; (**B**) body weight change curve; (**C**) DAI score curve; (**D**) body weight reduction in each group on day 21; (**E**) DAI score in each group on day 21; (**F**) colon length; and (**G**) representative images of the colon of mice in each group are shown. (*n* = 8) ** *p* < 0.01, *** *p* < 0.001, **** *p* < 0.0001.

To clarify the impact of dietary HP supplementation on colitis symptoms, a mouse model of UC caused by DSS was used. The experimental procedure is shown in Figure 3A. Compared with those in the NC group, DSS-treated mice had lower body weights (Figure 3B,D), higher DAI scores (Figure 3C,E), and shorter colons (Figure 3F,G); however, HP intervention relieved these symptoms, which indicated that HP reduced DSS-induced colitis.

### 3.3. HP Inhibits DSS-Induced Inflammation and Oxidative Stress

**Figure 4:**
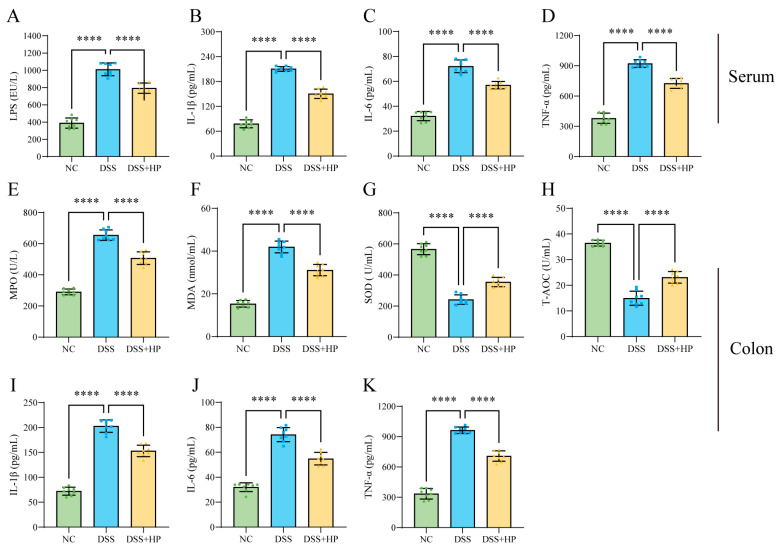
HP inhibits DSS-induced inflammation and oxidative stress. (**A**–**D**) ELISAs were performed to determine the serum levels of LPS, IL-1β, IL-6, and TNF-α. (**E**–**K**) ELISAs were performed to determine the levels of MPO, MDA, SOD, T-AOC, IL-1β, IL-6, and TNF-α in colon tissue. (*n* = 8) **** *p* < 0.0001.

The effects of HP intervention on DSS-induced oxidative stress and inflammation were examined by determining the serum and colon tissue levels of lipopolysaccharide (LPS), myeloperoxidase (MPO), proinflammatory factors (IL-6, IL-1β, and TNF-α), and oxidative stress-related indices (MDA, SOD, and T-AOC) via an ELISA. The assay data indicated that the serum levels of LPS and proinflammatory factors (IL-6, IL-1β, and TNF-α) were significantly greater (*p* < 0.0001) after DSS induction (Figure 4A–D); significantly increased amounts of MDA, MPO, and three proinflammatory markers were found in colon tissues (*p* < 0.0001) (Figure 4E,F,I–K). Furthermore, there was a noticeably reduced concentration of the antioxidant factors SOD and T-AOC (*p* < 0.0001) (Figure 4G,H). In contrast, HP intervention significantly alleviated the oxidative stress and inflammation brought on by DSS.

### 3.4. HP Alleviates DSS-Induced Damage to the Gut Barrier 

**Figure 5:**
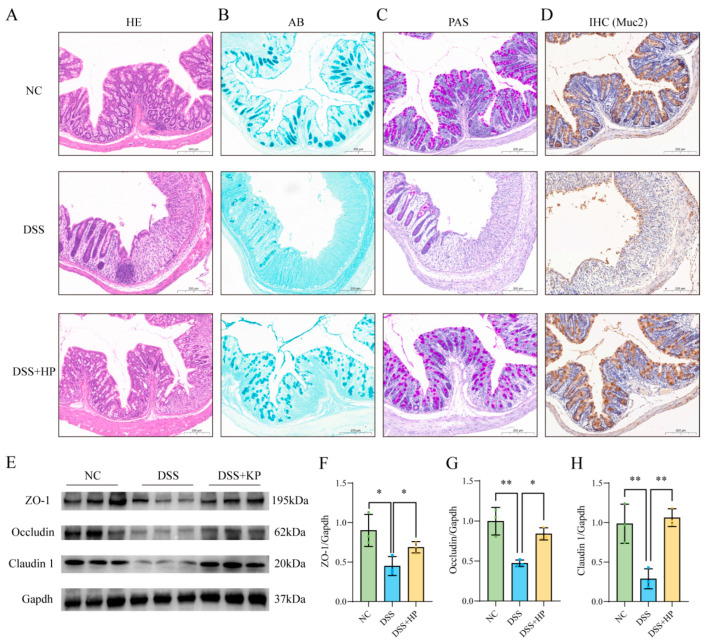
HP alleviates DSS-induced intestinal mechanical barrier damage. (**A**) HE staining of colon tissue sections; (**B**) AB staining; and (**C**) PAS staining were performed. (**D**) Immunohistochemical detection of Muc2 protein expression is shown. (**E**) Western blotting was utilised to assess tight junction protein (ZO-1, Occludin, Claudin 1) expression. (**F**–**H**) Quantitative analysis of ZO-1, Occludin, and Claudin 1 protein expression was performed. (*n* = 8) * *p* < 0.05, ** *p* < 0.01.

The body’s first line of defence against dangerous chemicals in the intestinal tract is the intestinal physical barrier, which is made up of tight junction proteins and mucus. Intestinal damage, the number of cup cells, and mucus secretion were analysed via pathological staining and immunohistochemistry. Colonic staining revealed inflammatory cell infiltration, mucosal oedema, and crypt damage after DSS induction (Figure 5A); there was also decreased mucus secretion (Figure 5B), loss of goblet cells (Figure 5C), and decreased Muc2 protein expression (Figure 5D). The HP intervention group showed a notable improvement in the DSS-induced injuries listed above when compared to the DSS group. The expression of ZO-1, Occludin, and Claudin 1 in the colon was determined via Western blotting. The results indicated that HP intervention significantly alleviated the DSS-induced decreases in tight junction protein expression (*p* < 0.05) (Figure 5E–H).

### 3.5. HP Alleviates DSS-Induced Gut Microbiota Disorder

**Figure 6:**
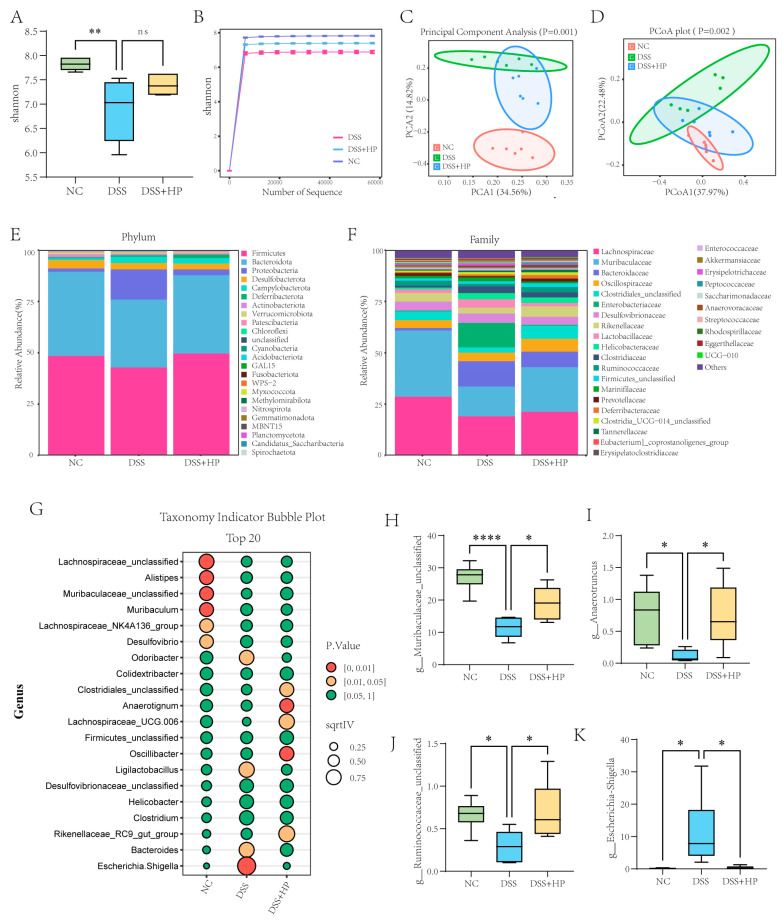
HP mitigated DSS-induced disruption of the gut microbiota. (**A**) Shannon’s index; (**B**) Shannon’s index sparse curve; (**C**) PCA; (**D**) PCoA; (**E**) species composition map at the phylum level; (**F**) species composition map at the family level; and (**G**) results of the indicative species analyses at the genus level are shown. (**H**–**K**) The abundance of *Muribaculaceae_unclassified*, *Anaerotruncus*, *Ruminococcaceae_unclassified*, and *Escherichia-Shigella* in each group was determined. (*n* = 8) * *p* < 0.05, ** *p* < 0.01, **** *p* < 0.0001, ns *p* > 0.05.

To assess the effect of HP on the gut microbiota, 16S rRNA sequencing was used to examine the microbiota composition of the caecal contents. Alpha diversity analysis revealed that the Shannon index decreased significantly after DSS treatment (*p* < 0.01), but HP intervention had no significant effect on the Shannon index (Figure 6A). The results of the dilution curve indicated that the sample size and sequencing depth were sufficient (Figure 6B). Principal component analysis (PCA) and principal coordinate analysis (PCoA) were used to examine the β diversity of the various samples. And the results indicated that the microbial composition of the three groups was different; the DSS group was distinctly separate from the NC group, whereas the DSS + HP group displayed a trend towards reverting back to the NC group (Figure 6C,D). Figure 5E,F show the gut microbial compositions of the three groups of mice at the phylum and family levels, respectively. Indicator analysis and linear discriminant analysis (LDA) effect size (LEfSe) analysis were widely used to reveal the characteristic different species between the groups. The results of the genus-level indicator analysis showed that *Alistipes*, *Desulfovibrio*, *Lachnospiraceae_NK4A136_group*, *Lachnospiraceae_unclassified*, *Muribaculaceae_unclassified*, and *Muribaculum* were associated with the NC group, and *Ligilactobacillus*, *Odoribacter*, *Bacteroides*, and *Escherichia-Shigella* were associated with the DSS group. *Clostridiales_unclassified*, *Anaerotignum*, *Lachnospiraceae_UCG-006*, *Oscillibacter*, and *Rikenellaceae_RC9_gut_group* were identified in the DSS + HP group (Figure 6G). In addition, HP intervention significantly reversed the DSS-induced decrease in the abundance of *Muribaculaceae_unclassified*, *Anaerotruncus*, and *Ruminococcaceae_unclassified* and increase in the abundance of *Escherichia-Shigella* (*p* < 0.05) (Figure 6H–K).

### 3.6. Effect of HP on Intestinal Metabolism

**Figure 7:**
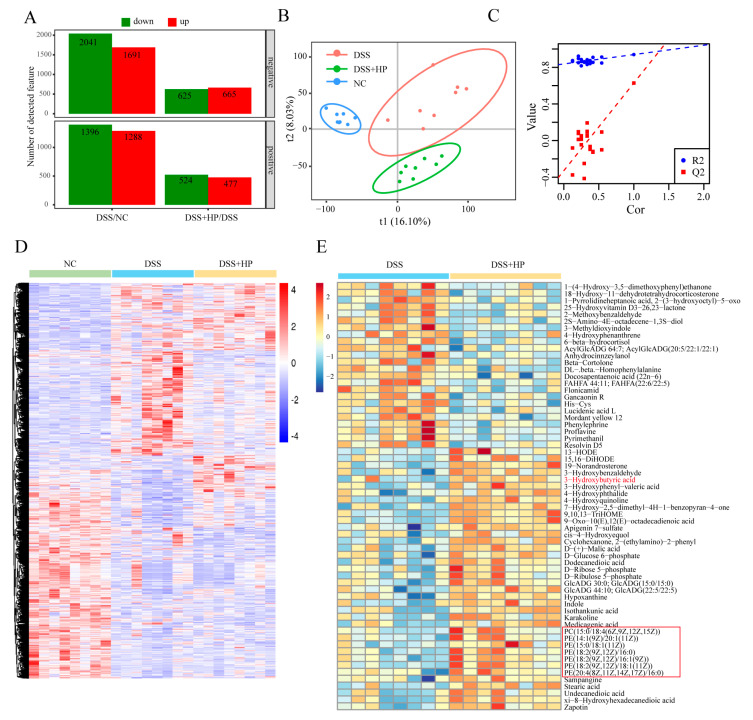
HP alleviates DSS-induced intestinal metabolic disorders. (**A**) Differentially abundant metabolites in the positive and negative ion modes were identified (fold change ≥ 1.5 or ≤1/1.5, *p* < 0.05, and VIP value ≥ 1). (**B**) PLS-DA and (**C**) results of the PLS-DA substitution test are shown. (**D**) Differentially abundant metabolite heatmaps among the three groups are shown. (**E**) The differential multiples of differences ≥2 or ≤0.5, *p* value < 0.05, and VIP value ≥ 2 thresholds were applied for differentially abundant metabolite heatmaps (*n* = 8).

To explore the effect of HP on intestinal metabolism, the cecum contents of mice were analysed by using nontargeted metabolomics. By applying the criteria of a fold change ≥1.5 or ≤2/3, *p* < 0.05, and a VIP value ≥1, a total of 2041 metabolites with downregulated expression were identified, whereas 1691 metabolites with upregulated expression were identified in the DSS group in comparison with the NC group in the negative ion mode. On the other hand, compared with the DSS group, there were 625 metabolites with downregulated expression and 665 metabolites with upregulated expression in the DSS + HP group. In the positive ion mode, the DSS group exhibited 1396 metabolites with downregulated expression and 1288 metabolites with upregulated expression in contrast to the NC. In the DSS + HP group, 524 metabolites had downregulated expression, and 477 metabolites had upregulated expression compared to the DSS group (Figure 7A). The partial least squares discriminant analysis (PLS-DA) technique was used for downregulation analysis, which demonstrated that the metabolic profiles of the three groups varied (Figure 7B), and the replacement test results demonstrated that the PLS-DA analysis did not lead to overfitting (Figure 7C). In Figure 7D, the clustering pattern of the metabolites heatmap with a different abundance was observed in the three groups of mice. Figure 7E shows the differentially abundant metabolites between the DSS and DSS-HP groups with a fold change ≥2 or ≤2/3, VIP value ≥2, and *p* < 0.05 in the form of a heatmap. Among them, the phosphatidylethanolamine and 3-hydroxybutyric acid levels were significantly higher after HP intervention.

### 3.7. Spearman Correlation Analysis Results

**Figure 8:**
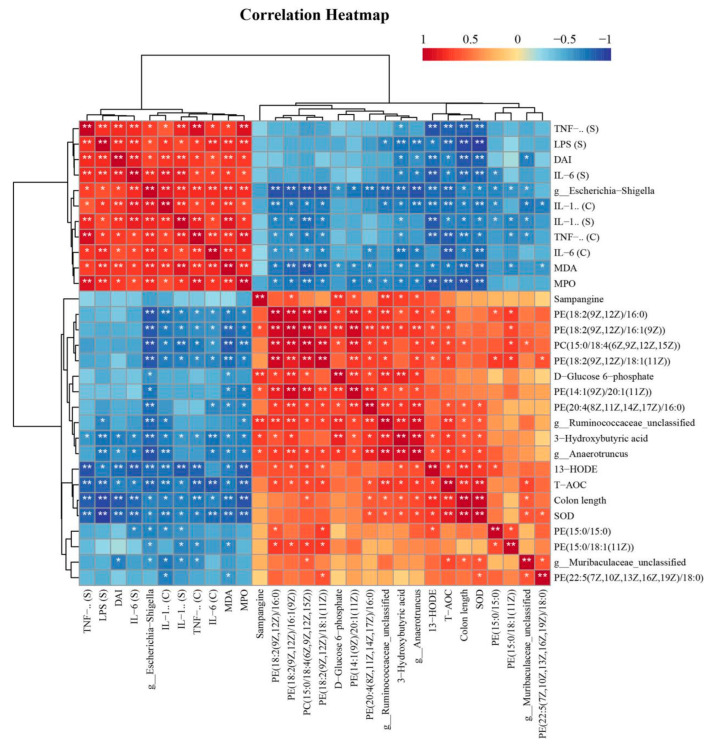
Spearman correlation analysis among differentially abundant microorganisms in the gut, differentially abundant metabolites in the gut, and biochemical indicators. (S) indicates serum samples, and (C) indicates colon tissue * *p* < 0.05, ** *p* < 0.01.

Spearman correlation analysis was used to examine the relationships between differentially abundant gut bacteria, differentially abundant gut metabolites, and biochemical markers. The results revealed that the abundance of *Escherichia-Shigella* was positively correlated with DAI score and inflammatory and oxidative stress indices, while it was negatively associated with colon length, differentially abundant metabolites, and antioxidant factors; the abundances of *Muribaculaceae_unclassified*, *Anaerotruncus*, and *Ruminococcaceae_unclassified* showed a favourable correlation with colon length, antioxidative factors, and differentially abundant metabolites and a negative inverse relationship with DAI scores, inflammatory, and oxidative indices (Figure 8).

## 4. Discussion

UC is a chronic inflammatory disease with an alternating relapsing–remitting course. UC cannot be cured, and the development of a dietary supplement for the prevention of UC is essential. Huaier has been used medicinally for more than 1000 years, and polysaccharides are among the main active substances in Huaier. And the DSS-induced mouse colitis model was the most widely used common model for studying acute colitis, which has the advantages of high credibility, simplicity, and wide applicability [24,33]. In the present study, HP significantly alleviated DSS-induced colitis-like symptoms, including weight loss, elevated DAI scores, and shortened colons. Mechanistically, HP may act by inhibiting oxidative stress and inflammation, helping maintain gut barrier integrity and regulating intestinal metabolism.

In colitis, oxidative stress and an escalation of the inflammatory response are major factors that lead to epithelial cell destruction [30,34]. It has been shown that Huaier polysaccharide (HP-1) can inhibit cisplatin-induced oxidative stress and apoptosis in the kidney through the PI3K-Akt pathway and also inhibit the expression of p-NF-κB to exert its anti-inflammatory function [35]. The outcomes of cellular studies in our investigation indicated that the pretreatment of HP significantly inhibited H2O2-induced oxidative stress and cellular damage. And our animal experiments confirmed that HP could effectively alleviate DSS-induced inflammatory responses and oxidative stress. We speculate that HP may reduce the expression of inflammatory mediators by inhibiting the NF-κB pathway, while enhancing cellular antioxidant defences by activating the PI3K/Akt pathway. Interestingly, in macrophages, the Huaier proteoglycan TPG-1 has been shown to enhance the immune response through the activation of the TLR4-NF-κB/MAPK pathway [36]. In addition, Yamin Li et al. demonstrated that the aqueous extracts of Huaier enhanced phagocytosis in macrophages [37]. These results suggest that Huaier polysaccharides have immunopotentiating effects and that their favourable antitumour activity may be due to immunopotentiation. We speculate that the reason for this difference in the results is due to the different cell lines selected and the differences in the active components of Huaier polysaccharides. Wang et al. found that the aqueous extract of Huaier was able to inhibit DSS-induced inflammation by suppressing the activation of NLRP3 inflammatory vesicles [22]. In the present study, HP treatment suppressed DSS-induced inflammation and oxidative stress. However, the specific signalling pathways mediated by HP and its interactions with specific receptors need to be further investigated.

The gut barrier components include the intestinal physical barrier (mucus layer and tight junction proteins), the immune barrier, and the microbial barrier. In this study, we analysed the effects of dietary supplementation with HP on the intestinal physical and microbial barriers. Dietary supplementation with HP helped maintain intestinal physical barrier integrity by promoting the expression of TJ proteins, maintaining the number of goblet cells, and promoting mucus secretion. 16S rRNA sequencing revealed that HP intervention significantly relieved the DSS-induced gut microbiota imbalance, as evidenced by increases in the abundance of *Muribaculaceae_unclassified*, *Anaerotruncus*, and *Ruminococcaceae_unclassified* and a decrease in the abundance of *Escherichia-Shigella*. Among them, *Muribaculaceae_unclassified* was a gut microorganism found in healthy people and was involved in butyrate metabolism and tryptophan metabolism [38]. Muribaculaceae can produce short-chain fatty acids, which are beneficial to the human body [39]. Interventions with fucoidan [40] and Nostoc commune Vaucher polysaccharide [41] were shown to increase the abundance of *Muribaculaceae_unclassified* and alleviate DSS-induced imbalance in the gut microbiota. According to Spearman correlation analysis, Muribaculaceae_unclassified was positively correlated with colon length, SOD, and T-AOC, while it was negatively correlated with DAI score and inflammatory and oxidative indices. Xiao et al. found that Anaerotruncus may be the best probiotic because it is a producer of butyric acid [42]. *Anaerotruncus*, a butyrate-producing bacterium, was positively correlated with intestinal sialylated spermine levels [43]. Bioinformatics analysis revealed a correlation between reduced *Anaerotruncus* abundance and diabetic nephropathy [44], endometriosis [45], and age-related macular degeneration [46]. Moreover, astaxanthin succinate diester intervention increased the abundance of *Anaerotruncus* in UC mice [47]. Our study showed that Anaerotruncus was positively correlated with colon length, SOD, and T-AOC, but it was negatively correlated with inflammatory markers, LPS, MDA, and MPO. The abundance of *Ruminococcaceae*, which is also a butyric-acid-producing probiotic, was significantly reduced in Crohn’s disease patients [48]. *Escherichia-Shigella*, which is a proinflammatory pathogen, was detected in IBD patients, and DSS-induced UC models exhibited an elevated abundance of *Escherichia-Shigella* [49,50]. Xu et al. found a positive correlation between Shigella and severity of inflammation and colitis, which is consistent with our study [51]. Supplementation with dietary additives such as sea cucumber and Artemisia ordosica crude polysaccharide inhibited the DSS-induced increase in the abundance of *Escherichia-Shigella* [52,53]. Thus, these results suggest that HP treatment improved the intestinal flora of mice by enhancing the abundance of beneficial bacteria and inhibiting harmful bacteria.

An essential part of the adaptive immune system are T cells. Th1, Th2, Th17, and regulatory T (Treg) cells are the four primary functional subtypes that arise from T cell activation by antigen-presenting cells. Specific gut microorganisms and functional genes influence the susceptibility of the body to colitis by regulating the ratio of Th cells. Recent studies have shown that ELF4 can alleviate colitis by inhibiting Th17 cell activity and promoting M2-type macrophage polarisation [54]; *Bacteroides uniformis*, *Bacteroides faecis*, *Faecalibacterium prausnitzii*, and *Roseburia intestinalis* attenuate DSS-induced ulcerative colitis by modulating the Treg/Th17 cell balance [55,56]. In asthma, huai qi huang granules containing Huaier plasmids have been shown to modulate ovalbumin-induced Th1/Th2 and Treg/Th17 cell imbalances [57]. However, the ability of HP to alleviate DSS-induced Th1/Th2 and Treg/Th17 cell imbalances still needs to be investigated.

Nontargeted metabolomics revealed significantly higher levels of 3-hydroxybutyric acid, phosphatidylcholine (PC), and phosphatidylethanolamine (PE) in the intestine after HP intervention. 3-Hydroxybutyric acid has various functions, including regulating blood pressure [58], inhibiting aging [59], and inhibiting colorectal cancer [60], and has recently been reported to prevent inflammatory bowel disease by increasing the proportion of regulatory T cells [61]. Two essential elements of eukaryotic membranes, PC and PE, are crucial for the maintenance of membrane integrity as well as the production of lipid droplets, autophagosome formation, and other physiological functions [62]. Dietary supplementation with phospholipids can treat inflammatory diseases and modulate inflammatory responses [63]. Therefore, we hypothesised that HP could restore gut barrier homeostasis by regulating intestinal metabolism and promoting the levels of various beneficial substances, such as 3-hydroxybutyric acid and phospholipids.

According to the body surface area normalisation method recommended by the US Food and Drug Administration [64] for interspecies drug dose conversion, the drug dose per unit body weight is usually higher in small animals than in humans, relative to body surface area [65]. In mice, the dosage is roughly 12.3 times higher than in humans [66]. In the present study, the HP dose administered by gavage to mice was 200 mg/kg, which we converted to a human dose of 16 mg/kg based on an estimate of 60 kg body weight per capita. Safety is a major concern in treatment. Huaier granules, a proprietary Chinese medicine, have been reported to have the advantages of low toxicity and ease of administration [67]. Yao et al. found that a clinically low risk of myelosuppression and hepatotoxicity occurred in patients with breast cancer using Sophora ear granules [68]. It is also noteworthy that mice given the previously specified 200 mg/kg dose did not experience any appreciable acute toxicity. However, future studies are needed to further fully evaluate its toxicology and side effects.

## 5. Conclusions

In conclusion, we presented evidence indicating that HP mitigated DSS-induced colitis through the maintenance of intestinal barrier integrity, modulation of the gut microbiota, and metabolic remodelling. These findings will contribute to a theoretical foundation for utilizing HP as a supplement to the diet to prevent UC. However, we only used the DSS-induced colitis model and the HT-29 cell line, and more experiments across different models are still needed to explore the protective effect of HP against colitis.

## Data Availability

The raw sequence data reported in this paper have been deposited in the Genome Sequence Archive in National Genomics Data Center, China National Center for Bioinformation/Beijing Institute of Genomics, Chinese Academy of Sciences (GSA: CRA013564, 3 August 2023), which are publicly accessible at https://bigd.big.ac.cn/gsa/browse/CRA013564 on 24 November 2023.
